# The impact of divergent forms of social support on health-related quality of life in patients with multiple myeloma and its precursor states

**DOI:** 10.1007/s00432-023-05570-9

**Published:** 2024-01-31

**Authors:** Anja Greinacher, Rea Kuehl, Elias K. Mai, Hartmut Goldschmidt, Joachim Wiskemann, Anna Fleischer, Leo Rasche, Ulrike Dapunt, Imad Maatouk

**Affiliations:** 1 Institute of Medical Psychology, Heidelberg University Hospital, Heidelberg, Germany; 2 Clinic for Palliative Medicine, Heidelberg University Hospital, Heidelberg, Germany; 3 National Center for Tumor Diseases, Department of Medical Oncology, Heidelberg University Hospital, Heidelberg, Germany; 4 Department of Internal Medicine V, Heidelberg University Hospital, Heidelberg, Germany; 5 National Center for Tumor Diseases (NCT) Heidelberg, Heidelberg, Germany; 6 Department of Internal Medicine II, Julius-Maximilian University Würzburg, Würzburg, Germany; 7 Department of General Internal Medicine and Psychosomatics, Heidelberg University Hospital, Heidelberg, Germany

**Keywords:** Multiple myeloma, Psychological distress, Health-related quality of life, Social support, Positive support, Detrimental interaction

## Abstract

**Purpose:**

Multiple myeloma is a largely incurable disease. Patients suffer from the cancer, therapeutic side effects, and often psychological symptoms. Not only multiple myeloma patients but also patients with precursor diseases show high psychological distress. Today, treatment option evaluations are increasingly performed in combination with health-related quality of life (HRQoL) assessments. One factor that is positively associated with HRQoL is social support.

**Methods:**

Our recent study used questionnaires (EORTC QLQ-C30, EORTC QLQ-MY20, Illness-specific Social Support Scale) to investigate the influence of positive and negative aspects of social support on HRQoL in patients with multiple myeloma and its precursors.

**Results:**

Multiple linear regression analyses with sex, age, treatment line, hemoglobin level, and number of comorbidities as control variables show that positive social support had a significant beneficial association with emotional function (*β* = 0.323) and social function (*β* = 0.251). Detrimental interactions had a significant negative association with social function (*β* =  − 0.209) and a significant positive association with side effects of treatment (*β* = 0.266).

**Conclusion:**

Therefore, screening for social support and, if needed, psycho-oncological care can be an important resource and should be implemented in routine care.

**Clinical trial registration:**

This study was registered with clinicaltrials.gov (NCT04328038).

## Introduction

Multiple myeloma (MM) is, in most patients, an incurable hematological malignancy from the B-cell lineage. The median age of patients at first diagnosis is 69 years (National Cancer Institute). MM is preceded by asymptomatic precursor conditions, including monoclonal gammopathy of undetermined significance (MGUS) and smoldering multiple myeloma (SMM) (Landgren et al. 2009). With the advent of novel therapeutic agents, including proteasome inhibitors (PIs, e.g., bortezomib), immunomodulatory agents (IMiDs, e.g., lenalidomide) and monoclonal antibodies (mAbs, e.g., daratumumab), life expectancy has constantly increased within the past decade. For example, in transplant-ineligible patients with newly diagnosed MM, five-year progression-free survival (PFS) is 52.5%, and the median overall survival (OS) has not been reached applying a therapy consisting of the anti-CD38 mAb daratumumab, lenalidomide and dexamethasone (Facon et al. 2021). This drives a paradigm shift in the MM landscape, with a need to limit the side effects of the therapy, prevent morbidity and mortality, and increase health-related quality of life (HRQoL). HRQoL in MM is impaired by disease-specific characteristics such as bone pain, anemia, and renal impairment but also psychological distress, e.g., fear of MM relapse, depressive symptoms and anxiety (Maatouk et al. 2019). A previous study showed that not only patients with MM but also patients with precursor diseases (MGUS and SMM) suffered from psychological distress above cutoff scores measured using the National Comprehensive Cancer Network distress thermometer (Maatouk et al. 2019).

As a result, HRQoL and lifestyle considerations have become more important in MM patients (Perrot et al. 2021; Shapiro et al. 2021). More recent trials increasingly include HRQoL as a key outcome (Mohyuddin et al. 2021), and from the patients’ perspective, prolonged PFS appears to be a goal equal to high HRQoL (Fleischer et al. 2021). A high HRQoL reflects both a lower symptom burden for patients and lower costs for the healthcare system. It has been shown that the psychological factors of HRQoL are independent prognostic factors for MM (Strasser‐Weippl and Ludwig 2008). In patients with various forms of cancer (Manne et al. 2015; Ristevska-Dimitrovska et al. 2015; Wu et al. 2015; Ye et al. 2017) but also in MM patients (Maatouk et al. 2018), HRQoL has been positively influenced by high resilience. One resilience factor that has been associated with HRQoL in MM patients is social support (Hu et al. 2021; Maher & De Vries 2011; Mortensen & Salomo 2016). Social support describes “perceived or objectively existing resources available to a person in his or her social network” (p. 2; Geue et al. 2019). Aside from positive support (PS), e.g., emotional, instrumental, esteem, and tangible support, there are also negative social interactions, i.e., detrimental interactions (DI), including omitting previously promised assistance, making critical remarks, suppressing expressions of emotion, overstepping personal boundaries and not protecting privacy (Lincoln 2000). The influence of DI has thus far only rarely been studied in cancer patients (Sauer et al. 2019), including hematological malignancies (Geue et al. 2019).

For these reasons, our current study aimed to investigate, for the first time, the association of positive and negative aspects of social support on the HRQoL of MM patients.

## Methods

### Study design and procedures

This cross-sectional survey was conducted from July to October 2020 at the National Center for Tumor Diseases (NCT) in Heidelberg, Germany. Eligible patients with MM and its precursor diseases were approached during their outpatient visits. They were asked to fill out the questionnaire immediately. If this was not possible, the questionnaire was completed at home and returned at the next appointment (*n* = 11, 7.9% of included patients). Patients younger than 18 years and patients who were scheduled for surgery within the next 24 weeks or whose last surgery was less than six weeks prior were excluded from the study.

### Ethical considerations

This study was approved by the ethics committee of the University Hospital Heidelberg (application no. S-875/2019). All participants provided written informed consent to participate and were able to withdraw their participation without any disadvantage. This study was conducted in accordance with the Declaration of Helsinki (most recent version; Fortaleza, Brazil, 2013) and registered with clinicaltrials.gov (NCT04328038).

### Survey instruments

Health-related quality of life (EORTC QLQ-C30 and EORTC QLQ-MY20). HRQoL was assessed with the 30-item questionnaire of the European Organization for Research (Aaronson et al. 1993) and an additional 20 items for myeloma patients (Cocks et al. 2007). The EORTC QLQ-C30 consists of a global health and HRQoL scale, five functional scales (physical, role, cognitive, emotional, and social), and three symptom scales (fatigue, nausea, and vomiting); dyspnea, appetite loss, sleep disturbance, constipation, diarrhea, and the financial impact of the illness are assessed via one item. The EORTC QLQ-MY20 consists of two functioning scales (body images assessed via one item and future perspective) and two symptom scales (disease symptoms and side effects of treatment). As only 13 patients answered the item on hair loss distress, we excluded this item from the analysis. All scores are transformed to range from 0 to 100; high scores equal high HRQoL (global health scale), high functioning (functional scales), and high symptoms (symptom scales). The questionnaire has been validated in patients with MM (Delforge et al. 2015).

Illness-specific Social Support Scale (ISSS). The subjective perception of PS and DI were surveyed with the short version (Ullrich & Mehnert 2010) of the Illness-specific Social Support Scale (Revenson and Schiaffino 1990). The sum of the two scales PS and DI range from 0 to 16; higher scores indicate higher levels of positive support and detrimental interactions. DI suggests, among other things, overprotectiveness and excessive optimism or pessimism (Ramm & Hasenbring 2003). The German version was validated in a cancer population (Ullrich & Mehnert 2010). Cronbach’s α ranged in our study from 0.70 to 0.82, which indicates acceptable to good internal consistency.

Patient medical and sociodemographic data were assessed from electronic medical records.

### Data analysis

All data were coded and analyzed using SPSS®-24 software (IBM Corp 2016) Raw data are displayed as the mean values and SD. The influence of social support (PS and DI) on HRQoL (global health, functioning scales, and symptom scales) was calculated with multiple linear regression analyses with sex, age, treatment line, hemoglobin score (Hb, surveyed on the day of the survey), and number of comorbidities as control variables. Prior to regression analyses, one outlier was removed from the dataset with the help of the Mahalanobis generalized distance (*α* = 0.001). Due to multiple testing of whether social support was related to HRQoL, the significance level was adjusted using the Bonferroni‒Holm correction.

## Results

### Participants

In total, 170 patients with MM or precursor diseases were approached. Ten of these patients met the exclusion criteria. Twenty patients declined participation in this study. Overall, 140 patients were enrolled. A total of 132 questionnaires were returned for a response rate of 94.3%, of which six (4.3%) were excluded due to low data quality. In total, 126 patients (90.0%) were included in the final analysis. A total of 42.5% (*n* = 54) of these patients were female, and the mean age was 64.10 years (SD = 9.50; range 38–84). Sociodemographic characteristics are displayed in Table 1.

**Table 1:** Sociodemographic data (*N* = 126)

	*n*	%	
Sex			
Male	72	56.7	
Female	54	42.5	
Age	*M* = 64.10	SD = 9.50	Range: 38–84
Family status			
Single	5	4.0	
Married/partnership	102	81.0	
Divorced/separated	13	10.2	
Widowed	5	4.0	
Missing	1	0.8	
Education			
No degree	1	0.8	
Secondary school	34	27.0	
Middle School	26	20.6	
High School	14	11.1	
University degree	50	39.7	
Missing	1	0.8	
Employment			
Yes	43	34.1	
No	72	57.1	
Missing	11	8.8	

### Disease characteristics and descriptive data

A total of 77.0% of the patients (*n* = 97) were diagnosed with MM, 15.0% with SMM (*n* = 19) and 8.0% with MGUS (*n* = 10). On average, participants were diagnosed four years before the study began (range 0–11). Thirty-five patients were in treatment line 0 (Rajkumar et al. 2015). Further information on disease characteristics is shown in Table 2. Mean values and standard deviation for all subscales of HRQoL and social support are displayed in Table 3.

**Table 2:** Disease characteristics (*N* = 126)

	*n*	%	
Diagnosis			
Multiple myeloma	97	77.0	
sMM	19	15.0	
MGUS	10	8.0	
Years since initial diagnosis	M = 4		Range: 11–0
Treatment line^a^			
0 (incl. precursor diseases and new diagnosis)	35	27.8	
1	65	51.6	
> 1	26	20.6	
Received treatment			
Chemotherapy	75	59.1	
Radiation	38	29.9	
Immunotherapy	48	37.8	
Autologous stem cell transplantation^b^	68	53.5	
Comorbidity			
Cardiovascular	52	41.3	
Pulmological	58	46.0	
Diabetes	2	1.6	
Other	72	57.1	
Number of comorbidities (categories)			
0	22	17.5	
1	48	38.1	
2	33	26.2	
3	22	17.5	
4	1	0.8	
Osteolyses^c^			
Yes	74	58.7	
No	52	41.3	
Hemoglobin^d^ g/dl	M = 12.80	SD = 1.46	Range: 8.8–16.4

^a^Based onRajkumar et al. (2015)

^b^After transplantation

^c^Taken from the medical documentation (radiology findings/physician ‘s medical report)

^d^Surveyed on the day of the questionnaire assessment

**Table 3:** Mean and standard deviation for EORTC QLQ-30, EORTC QLQ-MY20, and SSUK-8

	*N*	*M*	SD
EORTC QLQ-C30			
Global health/HRQoL	125	62.80	22.24
Physical Function	126	77.59	23.16
Role function	126	69.18	31.49
Emotional function	126	60.54	25.84
Cognitive function	126	75.79	25.53
Social function	125	64.80	31.55
Fatigue	126	43.30	28.43
Nausea/vomiting	125	6.53	13.86
Pain	126	35.58	31.29
Dyspnea	124	29.03	34.26
Insomnia	125	38.67	34.24
Appetite loss	126	13.76	26.42
Constipation	125	14.93	26.93
Diarrhea	126	19.05	30.24
Financial problems	125	17.33	29.81
EORTC QLQ-MY20			
Disease symptoms	125	27.62	22.33
Side effects of treatment	125	27.07	19.02
Body image	124	81.18	31.01
Future perspektive	125	4791	30.01
ISSS			
PS	118	13.40	3.16
DI	118	4.45	3.59

### Association of social support with health-related quality of life

Regression analyses with PS and DI as independent variables as well as sex, age, treatment line, Hb levels, and number of comorbidities as control variables showed a significant relationship with side effects of treatment (EORTC QLQ-MY20), emotional function (EORTC QLQ-C30), and social funtion (EORTC QLQ-C30). PS had a significant positive association with emotional function (*β* = 0.323) and social function (*β* = 0.251). DI had a significant negative association with social function (*β* =  − 0.209) and a positive association with side effects of treatment (*β* = 0.266). All regression models are found in Table 4.

**Table 4:** Regression analyses

Outcome variable	*N*	Predictors	*β*	*p*	*R*^2^		*p*	*p* value Bonferroni‒Holm correction
EORTC QLQ-MY20: side effects of treatment	117	Sex	– 0.126	0.158	0.219	*F*(7,109) = 4.360	< 0.001	0.01*
		Age	– 0.074	0.407				
		Treatment line	0.272	0.003				
		Hb	– 0.036	0.689				
		No. comorbidities	0.112	0.198				
		ISSS_PS	– 0.017	0.849				
		ISSS_DI	0.266	0.004				
EORTC QLQ-C30: emotional function	117	Sex	– 0.025	0.781				
		Age	0.160	0.082				
		Treatment line	– 0.106	0.258	0.179	*F*(7,109) = 3.388	0.003	0.027*
		Hb	0.091	0.326				
		No. comorbidities	– 0.010	0.908				
		ISSS_PS	0.323	< 0.001				
		ISSS_DI	– 0.174	0.062				
EORTC QLQ-C30: social function	116	Sex	– 0.030	0.743				
		Age	0.165	0.075				
		Treatment line	– 0.079	0.407	0.163	*F*(7,108) = 3.015	0.006	0.048*
		Hb ^a^	0.053	0.568				
		No. comorbidities	– 0.143	0.115				
		ISSS_PS	0.251	0.007				
		ISSS_DI	– 0.209	0.027				
EORTC QLQ-C30: cognitive function	117	Sex	0.126	0.176				
		Age	0.075	0.417		*F*(7,109) = 2.864		
		Treatment line	– 0.187	0.050	0.155		0.009	0.063
		Hb	0.055	0.556				
		No. comorbidities	– 0.054	0.552				
		ISSS_PS	0.222	0.016				
		ISSS_DI	– 0.121	0.200				
EORTC QLQ-C30: role function	117	Sex	– 0.033	0.725				
		Age	0.081	0.385				
		Treatment line	– 0.086	0.366	0.154	*F*(7,109) = 8.829	0.010	0.063
		Hb	0.146	0.122				
		No. comorbidities	– 0.167	0.065				
		ISSS_PS	0.227	0.014				
		ISSS_DI	– 0.156	0.099				
EORTC QLQ-C30: global health/HRQoL	116	Sex	– 0.052	0.576				
		Age	– 0.022	0.813		*F*(7,108) = 2.707		
		Treatment line	– 0.129	0.180	0.149		0.013	0.065
		Hb	0.207	0.030				
		No. comorbidities	– 0.033	0.715				
		ISSS_PS	0.174	0.059				
		ISSS_DI	– 0.177	0.063				
EORTC QLQ-C30: physical function	117	Sex	0.098	0.297				
		Age	– 0.076	0.416				
		Treatment line	– 0.101	0.295	0.135	*F*(7,109) = 2.420	0.024	0.096
		Hb	0.088	0.356				
		No. comorbidities	– 0.155	0.091				
		ISSS_PS	0.208	0.025				
		ISSS_DI	– 0.040	0.671				
EORTC QLQ-MY20: disease symptoms	117	Sex	– 0.226	0.018	0.130	*F*(7,109) = 2.320	0.030	0.096
		Age	– 0.012	0.899				
		Treatment line	0.113	0.241				
		Hb	– 0.055	0.566				
		No. comorbidities	0.037	0.685				
		ISSS_PS	– 0.041	0.660				
		ISSS_DI	0.196	0.042				
EORTC QLQ-MY20: body image	117	Sex	0.104	0.276	0.105	*F*(7,109) = 1.830	0.089	0.178
		Age	0.247	0.011				
		Treatment line	– 0.105	0.284				
		Hb	– 0.064	0.507				
		No. comorbidities	– 0.149	0.110				
		ISSS_PS	0.102	0.276				
		ISSS_DI	– 0.053	0.581				
EORTC QLQ-MY20: future perspektive	117	Sex	– 0.043	0.652	0.086	*F*(7,109) = 1.473	0.184	0.184
		Age	0.114	0.236				
		Treatment line	– 0.136	0.168				
		Hb	0.158	0.107				
		No. comorbidities	– 0.091	0.332				
		ISSS_PS	0.046	0.626				
		ISSS_DI	– 0.096	0.325				

^a^g/dl, surveyed on the day of the questionnaire assessment; low/weak variance explanation: *R*^2^ = 0.02, medium/moderate variance explanation: *R*^2^ = 0.13, high/strong variance explanation: *R*^2^ = 0.26, (Cohen 1988)

## Discussion

This cross-sectional study examined for the first time the association of the diverging forms of social support (PS and DI) on HRQoL (global health, function scales, and symptom scales) in patients with MM and precursor diseases. Sex, age, treatment line, Hb level, and number of comorbidities were included as control variables. PS had a significant positive association with emotional function and social function. DI had a significant positive association with the side effects of treatment and a significant negative association with social functioning.

The significant association of social support with HRQoL is consistent with previous research regarding patients with other cancers (Mehnert et al. 2010; Sauer et al. 2019; Soares et al. 2013). Previous studies have shown that PS and DI represent distinct constructs and do not belong to the same factor (Sauer et al. 2019). It should be emphasized that our study population reported PS more frequently than DI. This could be related to the higher average age of our study population. A study by Due et al. (1999) found an age difference in the structure and function of social relationships. The authors interpreted the results to reflect that people build social networks over their lifetimes in which PS predominates.

It can be concluded from the results that PS should be increased and DI should be reduced to improve HRQoL. Especially in the period around diagnostics, the PS of cancer patients seems to be of great importance. Studies suggest that PS reduces reoccurrence anxiety (Koch-Gallenkamp et al. 2016) and strengthens patients’ sense of coherence (Pasek et al. 2017). Furthermore, it predicts depression (Akechi et al. 2004; Eom et al. 2013; Hughes et al. 2014), anxiety (Ng et al. 2015), and distress (Akechi et al. 2006). A previous study by our research group (Sauer et al. 2019) showed that patients receive a similar pattern of social support in the year after diagnosis as they did at the time of receiving the diagnosis. The authors suggest that, in particular, patients with low PS or high DI should be identified as early as possible and offered help in the form of psycho-oncological care. Furthermore, other previous studies suggest that social support has an impact on somatic factors such as cancer progression, mortality (Frick et al. 2005), and inflammation levels (Hughes et al. 2014). For this reason, it may be helpful to screen cancer patients for social support as part of the diagnostic process. Patients with low PS and high DI can be offered support services to reinforce the positive aspects of social support and thus serve as an important resource. Interventions can include examining (e.g., with the CCAT-PF; Siminoff et al. 2008) and training communication patterns in couples and families (Zaider et al. 2017) or finding a better way to deal with DI. It should be considered that patients with MM are mostly elderly people. In contrast to younger people, they tend to be less oriented to the outer world and the future but more focused on internal processes and the present (Blank & Bellizzi 2008). On the one hand, this means that elderly people more often deal pragmatically with interpersonal conflicts, but on the other hand, it also implies that they tend to have fewer social contacts than younger people (Due et al. 1999). For this reason, interventions should pay attention to older people who do not have family (anymore) or who are only poorly socially integrated. Regular contacts (with psycho-oncological or social work interventions) can also provide basic stabilizing social support (Abbey et al. 1985). This topic has become particularly important in the current COVID-19 pandemic, as older people with preexisting illnesses were required to limit their contact with others.

### Strengths and limitations

To the best of our knowledge, this is the first study to investigate the association of social support (PS and DI) and HRQoL in MM patients. The strengths of this study are the subdivision of positive and negative aspects of social support as well as the differentially assessed control variables. Nevertheless, the limitations of the study should also be described: The study participants were patients in outpatient treatment. Thus, this is a patient group with comparatively mild symptoms and, in part, without treatment histories. For this reason, the study results cannot be generalized to patients in inpatient treatment. The results refer to cross-sectional data. For this reason, the relationship between social support and HRQoL must be interpreted with caution. The potential impact of social support on HRQoL needs to be replicated in future studies with a longitudinal design.

In summary, social support has a significant association with different domains of HRQoL in MM patients. Based on previous research, it can be assumed that social support can also influence the course of the disease. Screening for social support as part of the diagnostic process, followed by psycho-oncological care if needed, can help promote PS as well as better management of DI. Especially for patients who are not well-integrated socially, low-threshold regular contacts (e.g., by a psycho-oncologist or social worker) can be a stabilizing intervention. In the future, the influence of social support on the HRQoL of MM patients should also be investigated in longitudinal studies. This would provide important information over the course of the disease.

## Data Availability

The datasets generated and/or analyzed during the current study are available from the corresponding author upon reasonable request.

## References

[CR1] Aaronson NK, Ahmedzai S, Bergman B, Bullinger M, Cull A, Duez NJ, Filiberti A, Flechtner H, Fleishman SB, de Haes JC (1993) The European Organization for Research and Treatment of Cancer QLQ-C30: a quality-of-life instrument for use in international clinical trials in oncology. JNCI J Natl Cancer Inst 85(5):365–376 8433390 10.1093/jnci/85.5.365

[CR2] Abbey A, Abramis DJ, Caplan RD (1985) Effects of different sources of social support and social conflict on emotional well-being. Basic Appl Soc Psychol 6(2):111–129

[CR3] Akechi T, Okuyama T, Sugawara Y, Nakano T, Shima Y, Uchitomi Y (2004) Major depression, adjustment disorders, and post-traumatic stress disorder in terminally ill cancer patients: associated and predictive factors. J Clin Oncol 22(10):1957–1965 15143090 10.1200/JCO.2004.08.149

[CR4] Akechi T, Okuyama T, Akizuki N, Azuma H, Sagawa R, Furukawa TA, Uchitomi Y (2006) Course of psychological distress and its predictors in advanced non-small cell lung cancer patients. Psycho-Oncology 15(6):463–473 16173112 10.1002/pon.975

[CR5] Blank TO, Bellizzi KM (2008) A gerontologic perspective on cancer and aging. Cancer 112(S11):2569–2576 18428204 10.1002/cncr.23444

[CR6] Cocks K, Cohen D, Wisløff F, Sezer O, Lee S, Hippe E, Gimsing P, Turesson I, Hajek R, Smith A (2007) An international field study of the reliability and validity of a disease-specific questionnaire module (the QLQ-MY20) in assessing the quality of life of patients with multiple myeloma. Eur J Cancer 43(11):1670–1678 17574838 10.1016/j.ejca.2007.04.022

[CR7] Cohen J (1988) Statistical power analysis for the behavioural sciences, 2nd edn. Lawrence Earlbaum, Mahwah

[CR8] Corp IBM (2016) IBM SPSS Statistics for Windows. In: (Version 24.0). IBM Corp, Armonk

[CR10] Delforge M, Minuk L, Eisenmann J-C, Arnulf B, Canepa L, Fragasso A, Leyvraz S, Langer C, Ezaydi Y, Vogl DT (2015) Health-related quality-of-life in patients with newly diagnosed multiple myeloma in the FIRST trial: lenalidomide plus low-dose dexamethasone versus melphalan, prednisone, thalidomide. Haematologica 100(6):826 25769541 10.3324/haematol.2014.120121 PMC4450629

[CR11] Due P, Holstein B, Lund R, Modvig J, Avlund K (1999) Social relations: network, support and relational strain. Soc Sci Med 48(5):661–673 10080366 10.1016/s0277-9536(98)00381-5

[CR12] Eom CS, Shin DW, Kim SY, Yang HK, Jo HS, Kweon SS, Kang YS, Kim JH, Cho BL, Park JH (2013) Impact of perceived social support on the mental health and health-related quality of life in cancer patients: results from a nationwide, multicenter survey in South Korea. Psychooncology 22(6):1283–1290 22833521 10.1002/pon.3133

[CR13] Facon T, Kumar SK, Plesner T, Orlowski RZ, Moreau P, Bahlis N, Basu S, Nahi H, Hulin C, Quach H (2021) Daratumumab, lenalidomide, and dexamethasone versus lenalidomide and dexamethasone alone in newly diagnosed multiple myeloma (MAIA): overall survival results from a randomised, open-label, phase 3 trial. Lancet Oncol 22(11):1582–1596 34655533 10.1016/S1470-2045(21)00466-6

[CR15] Fleischer A, Heimeshoff L, Allgaier J, Jordan K, Gelbrich G, Pryss R, Schobel J, Einsele H, Kortuem M, Maatouk I (2021) Is PFS the right endpoint to assess outcome of maintenance studies in multiple myeloma? Results of a patient survey highlight quality-of-life as an equally important outcome measure. Blood 138:83634115103

[CR16] Frick E, Motzke C, Fischer N, Busch R, Bumeder I (2005) Is perceived social support a predictor of survival for patients undergoing autologous peripheral blood stem cell transplantation? Psycho-Oncology 14(9):759–770 15744779 10.1002/pon.908

[CR17] Geue K, Götze H, Friedrich M, Leuteritz K, Mehnert-Theuerkauf A, Sender A, Stöbel-Richter Y, Köhler N (2019) Perceived social support and associations with health-related quality of life in young versus older adult patients with haematological malignancies. Health Qual Life Outcomes 17(1):1–10 31438983 10.1186/s12955-019-1202-1 PMC6704656

[CR19] Hu X, Wang W, Wang Y, Liu K (2021) Fear of cancer recurrence in patients with multiple myeloma: prevalence and predictors based on a family model analysis. Psychooncology 30(2):176–184 32931646 10.1002/pon.5546 PMC7984278

[CR20] Hughes S, Jaremka LM, Alfano CM, Glaser R, Povoski SP, Lipari AM, Agnese DM, Farrar WB, Yee LD, Carson WE III (2014) Social support predicts inflammation, pain, and depressive symptoms: longitudinal relationships among breast cancer survivors. Psychoneuroendocrinology 42:38–44 24636499 10.1016/j.psyneuen.2013.12.016 PMC3970938

[CR23] Koch-Gallenkamp L, Bertram H, Eberle A, Holleczek B, Schmid-Höpfner S, Waldmann A, Zeissig SR, Brenner H, Arndt V (2016) Fear of recurrence in long-term cancer survivors—do cancer type, sex, time since diagnosis, and social support matter? Health Psychol 35(12):1329 27175578 10.1037/hea0000374

[CR24] Landgren O, Kyle RA, Pfeiffer RM, Katzmann JA, Caporaso NE, Hayes RB, Dispenzieri A, Kumar S, Clark RJ, Baris D (2009) Monoclonal gammopathy of undetermined significance (MGUS) consistently precedes multiple myeloma: a prospective study. Blood 113(22):5412–5417 19179464 10.1182/blood-2008-12-194241 PMC2689042

[CR25] Lincoln KD (2000) Social support, negative social interactions, and psychological well-being. Soc Serv Rev 74(2):231–252 26594064 10.1086/514478 PMC4651456

[CR26] Maatouk I, He S, Becker N, Hummel M, Hemmer S, Hillengass M, Goldschmidt H, Hartmann M, Schellberg D, Herzog W (2018) Association of resilience with health-related quality of life and depression in multiple myeloma and its precursors: results of a German cross-sectional study. BMJ Open 8(7):e021376 30061438 10.1136/bmjopen-2017-021376 PMC6067407

[CR27] Maatouk I, He S, Hummel M, Hemmer S, Hillengass M, Goldschmidt H, Hartmann M, Herzog W, Hillengass J (2019) Patients with precursor disease exhibit similar psychological distress and mental HRQOL as patients with active myeloma. Blood Cancer J 9(2):1–4 10.1038/s41408-019-0172-1 PMC6341083 30664623

[CR28] Maher K, De Vries K (2011) An exploration of the lived experiences of individuals with relapsed multiple myeloma. Eur J Cancer Care 20(2):267–275 10.1111/j.1365-2354.2010.01234.x 20950368

[CR29] Manne S, Myers-Virtue S, Kashy D, Ozga M, Kissane D, Heckman C, Rubin SC, Rosenblum N (2015) Resilience, positive coping, and quality of life among women newly diagnosed with gynecological cancers. Cancer Nurs 38(5):375 25521911 10.1097/NCC.0000000000000215 PMC4470889

[CR30] Mehnert A, Lehmann C, Graefen M, Huland H, Koch U (2010) Depression, anxiety, post-traumatic stress disorder and health-related quality of life and its association with social support in ambulatory prostate cancer patients. Eur J Cancer Care 19(6):736–745 10.1111/j.1365-2354.2009.01117.x 19832893

[CR31] Mohyuddin GR, Koehn K, Abdallah AO, Sborov D, Rajkumar SV, Kumar S, McClune B (2021) Use of endpoints in multiple myeloma randomized controlled trials over the last fifteen years: a systematic review. Am J Hematol. 96(6):690–697 33735453 10.1002/ajh.26166

[CR32] Mortensen G, Salomo M (2016) Quality of life in patients with multiple myeloma: a qualitative study. J Cancer Sci Ther 8:289–293

[CR33] Ng CG, Mohamed S, See MH, Harun F, Dahlui M, Sulaiman AH, Zainal NZ, Taib NA (2015) Anxiety, depression, perceived social support and quality of life in Malaysian breast cancer patients: a 1-year prospective study. Health Qual Life Outcomes 13(1):1–9 26715073 10.1186/s12955-015-0401-7 PMC4696207

[CR35] Pasek M, Dębska G, Wojtyna E (2017) Perceived social support and the sense of coherence in patient–caregiver dyad versus acceptance of illness in cancer patients. J Clin Nurs 26(23–24):4985–4993 28793381 10.1111/jocn.13997

[CR36] Perrot A, Facon T, Plesner T, Usmani SZ, Kumar S, Bahlis NJ, Hulin C, Orlowski RZ, Nahi H, Mollee P (2021) Health-related quality of life in transplant-ineligible patients with newly diagnosed multiple myeloma: Findings from the phase III MAIA trial. J Clin Oncol 39(3):227–237 33326255 10.1200/JCO.20.01370 PMC8078427

[CR37] Rajkumar SV, Richardson P, San Miguel JF (2015) Guidelines for determination of the number of prior lines of therapy in multiple myeloma. Blood 126(7):921–922 26272048 10.1182/blood-2015-05-647636

[CR38] Ramm GC, Hasenbring M (2003) Die deutsche Adaptation der Illness-specific Social Support Scale und ihre teststatistische Überprüfung beim Einsatz an Patienten vor und nach Knochenmarktransplantation. Z Med Psychol 12(1):29–38

[CR39] Revenson TA, Schiaffino K (1990) Development of a contextual social support measure for use with arthritis populations. In: Annual convention of the Arthritis Health Professions Association, Seattle, WA

[CR40] Ristevska-Dimitrovska G, Filov I, Rajchanovska D, Stefanovski P, Dejanova B (2015) Resilience and quality of life in breast cancer patients. Open Access Maced J Med Sci 3(4):727 27275317 10.3889/oamjms.2015.128 PMC4877917

[CR42] Sauer C, Weis J, Faller H, Junne F, Hönig K, Bergelt C, Hornemann B, Stein B, Teufel M, Goerling U (2019) Impact of social support on psychosocial symptoms and quality of life in cancer patients: results of a multilevel model approach from a longitudinal multicenter study. Acta Oncol 58(9):1298–1306 31284793 10.1080/0284186X.2019.1631471

[CR43] Shapiro YN, Peppercorn JM, Yee AJ, Branagan AR, Raje NS, Donnell EK (2021) Lifestyle considerations in multiple myeloma. Blood Cancer J 11(10):1–9 34702799 10.1038/s41408-021-00560-x PMC8548591

[CR44] Siminoff LA, Zyzanski SJ, Rose JH, Zhang AY (2008) The cancer communication assessment tool for patients and families (CCAT-PF): a new measure. Psychooncology 17(12):1216–1224 18504807 10.1002/pon.1350 PMC2830149

[CR45] Soares A, Biasoli I, Scheliga A, Baptista R, Brabo E, Morais J, Werneck G, Spector N (2013) Association of social network and social support with health-related quality of life and fatigue in long-term survivors of Hodgkin lymphoma. Support Care Cancer 21(8):2153–2159 23475196 10.1007/s00520-013-1775-x

[CR46] Strasser-Weippl K, Ludwig H (2008) Psychosocial QOL is an independent predictor of overall survival in newly diagnosed patients with multiple myeloma. Eur J Haematol 81(5):374–379 18637124 10.1111/j.1600-0609.2008.01126.x

[CR47] Ullrich A, Mehnert A (2010) Psychometrische evaluation and validierung einer 8-Item Kurzversion der Skalen zur Sozialen Unterstützung bei Krankheit (SSUK) bei Krebspatienten. Klinische Diagnostik Und Eval 3(4):359–381

[CR48] Wu W-W, Tsai S-Y, Liang S-Y, Liu C-Y, Jou S-T, Berry DL (2015) The mediating role of resilience on quality of life and cancer symptom distress in adolescent patients with cancer. J Pediatr Oncol Nurs 32(5):304–313 25612835 10.1177/1043454214563758

[CR49] Ye ZJ, Qiu HZ, Li PF, Liang MZ, Zhu YF, Zeng Z, Hu GY, Wang SN, Quan XM (2017) Predicting changes in quality of life and emotional distress in Chinese patients with lung, gastric, and colon-rectal cancer diagnoses: the role of psychological resilience. Psychooncology 26(6):829–835 27479936 10.1002/pon.4237

[CR50] Zaider T, Hichenberg S, Latella L, Kissane D, Bultz B, Butow P (2017) Advancing family communication skills in oncology nursing. Oxford Textbook of Communication in Oncology and Palliative Care, Oxford, p 181

